# The microbiome-gerogene axis: a new frontier in precision geromedicine

**DOI:** 10.3389/fragi.2026.1794192

**Published:** 2026-05-08

**Authors:** Jhommara Bautista, Andrés López-Cortés

**Affiliations:** Cancer Research Group (CRG), Faculty of Medicine, Universidad de Las Américas, Quito, Ecuador

**Keywords:** gerogenes, gut microbiome, hallmarks of aging, immunosenescence, precision geromedicine

## Abstract

Aging is increasingly recognized as a biologically heterogeneous process arising from dynamic interactions among genetic programs, environmental exposures, and adaptive physiological responses. Within the geroscience framework, conserved hallmarks, including genomic instability, epigenetic alterations, mitochondrial dysfunction, chronic inflammation, cellular senescence, and dysbiosis, capture the systems-level nature of age-related decline. Parallel to this framework, the concept of *gerogenes* defines coordinated molecular programs that actively drive biological aging when persistently engaged, counterbalanced by gerosuppressive pathways that preserve resilience. Here, we synthesize evidence supporting a unifying microbiome-gerogene axis in which the gut microbiome functions as an upstream, modifiable regulator of molecular aging trajectories. Age-associated microbial remodeling leads to loss of beneficial metabolic functions, including short-chain fatty acid production, bile acid transformation, and mitochondrial-supportive co-metabolism, with downstream effects on epithelial barrier integrity, immune homeostasis, and tissue repair. Integrated multi-omics studies link these microbial changes to host transcriptional, epigenomic, proteomic, and metabolomic signatures of biological aging, enabling mechanistic insights beyond taxonomic associations. Immune aging represents a major convergence point of microbiome-gerogene crosstalk, as dysbiosis driven barrier dysfunction and microbial translocation reinforce inflammaging, immunosenescence, and senescence-associated signaling networks. In parallel, microbial metabolites interface with epigenetic regulation, mitochondrial quality control, circadian biology, and gut-brain-immune communication, extending microbial influence to systemic and neurodegenerative aging processes. Building on this mechanistic foundation, we propose the microbiome-gerogene axis as an integrative framework for precision geromedicine, linking lifestyle exposures to intracellular aging programs and informing biomarker discovery and personalized interventions aimed at extending healthspan rather than treating late-stage disease.

## Introduction

Aging is increasingly recognized as a biologically heterogeneous process shaped by the dynamic interplay between genetic programs, environmental exposures, and adaptive physiological responses. Geroscience has provided a conceptual framework to understand aging through conserved molecular and cellular hallmarks, including genomic instability, epigenetic alterations, mitochondrial dysfunction, chronic inflammation, cellular senescence, and dysbiosis. Within this framework, the concept of gerogenes and signaling pathways that actively drive biological aging when overactivated, has emerged as a central explanatory model, analogous to oncogenes in cancer biology. Conversely, gerosuppressors counterbalance these processes, collectively determining individual aging trajectories and susceptibility to age-related diseases (Kroemer et al., 2025a; López-Otín et al., 2023a; Ghosh et al., 2022). In this context, gerogenes can be operationally defined as conserved molecular hubs and signaling programs, rather than isolated genes, that integrate metabolic, inflammatory, and stress-related inputs to modulate the pace of biological aging. These programs are increasingly quantifiable through coordinated transcriptomic, epigenomic, proteomic, and metabolomic signatures, enabling comparative assessment across tissues, physiological states, and life stages (Landsberger et al., 2024; López-Otín et al., 2024).

A clear distinction must be established between gerogenes and conventional aging-associated genes or pathways. Established frameworks, such as the hallmarks of aging, describe conserved biological processes that deteriorate over time, including genomic instability, mitochondrial dysfunction, and cellular senescence, defining discrete mechanisms or phenotypic endpoints without capturing coordinated regulatory control across systems. In contrast, gerogenes are conceptualized as higher-order regulatory programs that integrate multiple molecular pathways across cellular contexts. Gerogenes do not correspond to individual genes or isolated signaling cascades, but to coordinated network states in which metabolic, inflammatory, and stress-response pathways converge to regulate downstream transcriptional, epigenetic, and metabolic outputs that shape the trajectory of biological aging. In this sense, gerogenes describe system-level organization of aging processes, analogous to oncogenic programs in cancer biology, where coordinated signaling states define disease behavior more accurately than single genetic alterations (López-Otín et al., 2023a; López-Otín et al., 2024).

Parallel to advances in geroscience, microbiome research has transformed our understanding of host biology by revealing humans as meta-organisms in which microbial genomes function as a dynamic adaptive genome. The gut microbiome, in particular, exerts pleiotropic effects on host metabolism, immune regulation, epithelial integrity, and neuroendocrine signaling. Accumulating evidence demonstrates that age-associated remodeling of the microbiome is not merely a consequence of aging but is associated with and may amplify multiple aging hallmarks, including inflammaging, immunosenescence, and metabolic decline (Kim and Benayoun, 2020; Bosco and Noti, 2021; Gyriki et al., 2025). Importantly, microbiome-host crosstalk is established early in life: the perinatal period represents a critical window in which maternal, environmental, and clinical factors shape neonatal microbial assembly with durable consequences for immune priming, metabolic programming, and neurodevelopment. Consistent with a life-course framework, neonatal microbial profiles have been associated with later-life health trajectories and are being explored as research biomarkers, although clinical validation remains pending (Bautista and López-Cortés, 2025c).

Recent studies integrating multi-omics approaches have begun to elucidate the molecular interfaces through which microbial communities influence host aging. Metagenomic and metabolomic analyses reveal that aging is associated with a decline in beneficial microbial functions, including short-chain fatty acid (SCFA) production, bile acid transformation, nucleotide biosynthesis, and mitochondrial-supportive metabolic networks. These functional losses correlate with downregulation of host pathways essential for epithelial barrier maintenance, stem cell function, and immune homeostasis, processes tightly linked to gerogene activation (Best et al., 2025; Jing et al., 2025). Importantly, experimental microbiota transfer from young to aged hosts partially restores these pathways and improves healthspan-related phenotypes, providing causal evidence that microbial signals can modulate the pace of biological aging (Aldriwesh et al., 2026).

The immune system represents a major convergence point of microbiome–gerogene interactions. Aging is characterized by immunosenescence and chronic low-grade inflammation, driven in part by gut barrier dysfunction and microbial translocation. Age-related dysbiosis promotes pro-inflammatory cytokine networks and alters hematopoietic and lymphoid niches, reinforcing gerogenic circuits associated with stem cell exhaustion and impaired tissue regeneration. Conversely, maintenance of a diverse and functionally resilient microbiome supports immune tolerance, limits systemic inflammation, and preserves adaptive immune capacity, highlighting the microbiome as a modifiable determinant of immune aging (Bosco and Noti, 2021; Jing et al., 2025).

Beyond immunity, the microbiome interfaces with central gerogenic pathways through metabolic and neuroendocrine axes. Microbial metabolites such as SCFAs, tryptophan derivatives, and secondary bile acids act as signaling molecules that influence epigenetic regulation, mitochondrial function, circadian rhythms, and neuronal signaling. Disruption of these metabolite networks during aging has been linked to cognitive decline, behavioral alterations, and impaired stress responses, underscoring the systemic reach of microbiome-driven gerogene modulation (Jing et al., 2024; López-Otín and Kroemer, 2025). Notably, circadian dysregulation, a recognized hallmark of aging, intersects with microbial rhythmicity and feeding-related zeitgebers, suggesting bidirectional feedback between the circadian clock, microbiome dynamics, and aging pathways (García Cobarro et al., 2025). Emerging evidence further extends this framework by demonstrating that gut-brain-immune communication plays a critical role in age-related neurodegenerative vulnerability. Microbiome-driven immune activation, barrier dysfunction, and altered microbial metabolite signaling have been implicated in exacerbating chronic neuroinflammation, proteostatic imbalance, and mitochondrial dysfunction, thereby linking systemic aging processes with neurodegenerative trajectories (Bautista et al., 2025a).

Converging lines of evidence motivate the conceptualization of a microbiome-gerogene axis, in which microbial composition and function are associated with modulation of molecular aging programs. To improve conceptual clarity, a distinction is required between statistical association and causal inference in microbiome–host interactions. Statistical association refers to correlations between microbial features and host phenotypes identified through observational or multi-omics analyses, without evidence of directionality or mechanistic involvement, and may reflect confounding factors, reverse causation, or shared environmental influences. In contrast, causal association requires experimental or longitudinal evidence demonstrating that modulation of the microbiome induces reproducible changes in host molecular pathways or physiological outcomes, supported by microbiota transfer experiments, targeted perturbation of microbial communities or metabolites, and mechanistic validation linking microbial products to defined host receptors and signaling pathways (Chigozie and Esimone, 2026; Nagpal et al., 2018; Monaghan et al., 2025).

The microbiome-gerogene axis provides a mechanistic bridge between environmental and lifestyle factors, diet, physical activity, psychosocial stress, circadian alignment, and the intracellular pathways that determine biological aging. From a translational perspective, such a framework aligns with the goals of precision geromedicine, which seeks to tailor interventions based on individual molecular profiles, exposome histories, and adaptive capacities (Kroemer et al., 2025a; Ghosh et al., 2022). In such a context, aging-associated neurodegenerative processes are increasingly recognized as active contributors to systemic tissue remodeling across the lifespan. Neurodegeneration-induced immune and metabolic reprogramming may contribute to the formation of peripheral inflammatory niches, reinforcing organism-wide aging phenotypes through microbiome-dependent mechanisms (Bautista et al., 2025c).

Importantly, microbiome-based strategies offer unique advantages for geromedicine. Unlike static genetic determinants, the microbiome is plastic and responsive to targeted interventions, including diet modulation, prebiotics, probiotics, postbiotics, and fecal microbiota transplantation. Emerging evidence suggests that such interventions can recalibrate gerogenic signaling networks, restore metabolic and immune homeostasis, and potentially delay the onset of age-related multimorbidity (Best et al., 2025; Jing et al., 2025). However, inter-individual variability, context-dependent effects, and methodological heterogeneity underscore the need for integrative, systems-level approaches to fully harness the therapeutic potential of the microbiome-gerogene axis (Van Hul and Cani, 2026; Liu et al., 2024).

In this review, we synthesize current evidence supporting the microbiome as a key regulator of gerogenes and aging trajectories. By integrating insights from geroscience, microbiome biology, and systems medicine, we propose the microbiome-gerogene axis as a mechanistically grounded integrative framework precision geromedicine, with implications for biomarker discovery, risk stratification, and the development of personalized interventions aimed at extending healthspan rather than merely treating age-related disease.

## Microbiome-driven regulation of gerogenes

Aging is increasingly recognized as a genetically regulated process modulated by environmental inputs, among which the gut microbiome has emerged as a central and dynamic regulator. Within the geroscience framework, *gerogenes*, defined as genes and molecular pathways that actively promote biological aging when overactivated, are not only shaped by intrinsic genetic programs but are also profoundly influenced by microbiome-derived signals. Accumulating evidence indicates that the microbiome acts as a transducer of dietary, metabolic, and inflammatory cues that converge on gerogenic and gerosuppressive pathways, thereby shaping the trajectory of biological aging and healthspan (Kroemer et al., 2025a; Dato et al., 2017).

One of the most robust mechanisms by which the microbiome regulates gerogenes is through microbial metabolite signaling. SCFAs, including acetate, propionate, and butyrate, are among the best-characterized microbiome-derived metabolites influencing aging-related gene networks. SCFAs modulate histone acetylation via inhibition of histone deacetylases (HDACs), leading to broad epigenetic effects on genes involved in inflammation, mitochondrial function, and cellular senescence. These epigenetic actions directly intersect with canonical gerogenic pathways, including NF-κB signaling, mTOR activity, and stress-response transcriptional programs, thereby linking microbial ecology to chromatin-level regulation of aging (Ghosh et al., 2022; Binyamin et al., 2025; Kane and Sinclair, 2019). In line with this framework, disease models demonstrate that microbiome linked inflammatory-metabolic signaling can converge on central regulatory nodes such as NF-κB and JAK/STAT, and can couple metabolite availability to growth-factor pathways (e.g., IGF-1 - MAPK/PI3K). When persistently engaged, these networks mirror systems-level signaling patterns associated with accelerated aging phenotypes, providing mechanistic plausibility for microbiome-driven amplification of gerogenic programs (Bautista et al., 2025b; Peng et al., 2020).

Beyond SCFAs, the aging microbiome shapes host gerogenes through alterations in amino acid, nucleotide, and lipid metabolism. Integrated multi-omics studies demonstrate that age-associated microbiome remodeling leads to reduced microbial support for host nucleotide biosynthesis and mitochondrial respiration, pathways essential for tissue maintenance and genomic stability (Best et al., 2025; Chen et al., 2023). This metabolic decline is accompanied by downregulation of host genes involved in epithelial barrier integrity, DNA repair, and cellular replication, hallmark processes tightly linked to gerogenic activation (Best et al., 2025).

Inflammation represents another critical axis connecting the microbiome to gerogene regulation. Age-related dysbiosis promotes chronic low-grade inflammation, or inflammaging, through increased intestinal permeability and translocation of microbial products. This persistent inflammatory milieu activates gerogenic signaling cascades, including JAK/STAT, NF-κB, and inflammasome pathways, which accelerate cellular senescence and immune dysfunction. Experimental depletion or remodeling of the aged microbiome reverses inflammatory gene expression profiles in the gut and immune tissues, highlighting a causal role for microbiota-dependent regulation of inflammation-linked gerogenes (Bosco and Noti, 2021; Jing et al., 2025).

Importantly, microbiome–gerogene interactions are bidirectional. Host genetic pathways governing barrier function, immune sensing, and metabolic signaling actively shape microbial composition and function. Intestine-specific genetic perturbations affecting antimicrobial peptide production, bile acid signaling, or tight junction integrity lead to predictable shifts in microbial ecology, favoring pathobiont expansion and loss of beneficial taxa (Dey, 2025; Simonson et al., 2025). These genetically driven microbial changes, in turn, reinforce gerogenic activation by amplifying inflammatory and metabolic stress signals, establishing a self-perpetuating feedback loop between host gerogenes and microbial dysbiosis (Dey, 2025).

Studies of long-lived individuals provide compelling evidence that specific microbiome configurations are associated with attenuation of gerogenic programs. Centenarians and exceptionally long-lived populations often exhibit preserved microbial diversity and enrichment of taxa linked to anti-inflammatory and metabolic homeostasis. These microbial signatures correlate with transcriptional profiles indicative of reduced immune activation, improved mitochondrial efficiency, and enhanced stress resilience, features consistent with suppression of gerogenic pathways and promotion of healthy aging (Aldriwesh et al., 2026; Dato et al., 2017; Pu et al., 2024). Together, these observations support a model in which the microbiome functions as a systems-level regulator of gerogenes, integrating environmental exposures with host genetic networks. Rather than acting solely as a passive correlate of aging, the microbiome is associated with modulation of epigenetic, metabolic, and immune pathways that define the pace of biological aging (Kroemer et al., 2025a; Ghosh et al., 2022). In parallel, organ-axis perspectives in gastrointestinal disease highlight that microbiota-derived metabolites, including SCFAs and bile acids, can reshape immune and epigenetic signaling networks, reinforcing the broader principle that microbial function, more than taxonomy alone, can operate upstream of host regulatory programs relevant to aging and age-related disease (Bautista et al., 2025e; Wang et al., 2023).

## Microbial metabolites as epigenetic and transcriptomic modulators of aging

Microbial metabolites constitute a critical molecular interface through which the gut microbiome exerts systemic control over host aging trajectories. Beyond their classical metabolic and immunomodulatory roles, these small molecules act as potent epigenetic and transcriptomic regulators, shaping gene expression programs linked to longevity, cellular senescence, and tissue homeostasis. Accumulating evidence indicates that age-associated alterations in microbiome composition and intestinal barrier integrity profoundly modify the circulating metabolite pool, thereby influencing epigenetic drift and transcriptional reprogramming across multiple organs (Dato et al., 2017; Wang et al., 2022). These observations align with the concept of epigenetic memory, whereby transient or chronic exposures can generate stable DNA methylation and histone-mark patterns that persist beyond the initiating stimulus and continue to shape gene expression trajectories (Bautista and Lopez-Cortes, 2025; Del, 2025).

Among microbial-derived metabolites, SCFAs, particularly butyrate and propionate, represent the most extensively characterized epigenetic modulators. Produced through bacterial fermentation of dietary fiber, SCFAs directly influence chromatin architecture via inhibition of HDACs and through their conversion into acyl-CoA intermediates that serve as substrates for non-canonical histone acylations. Genome-wide mapping studies have demonstrated that propionylation and butyrylation of specific histone lysine residues increase chromatin accessibility at loci governing cell-cycle control, differentiation, stress responses, and metabolic regulation, thereby establishing a direct mechanistic link between microbial metabolism and host transcriptional landscapes (Nshanian et al., 2025; Furusawa et al., 2013). Epigenetic modulation driven by microbial and host-derived signals exhibits strong context dependence, supporting homeostatic gene expression in healthy tissues while restricting aberrant proliferative programs under pathological conditions or during aging-associated decline (Li and Tay, 2026). From a mechanistic perspective, these epigenetic effects exhibit strong tissue specificity and temporal dependence, underscoring that microbial metabolite-driven chromatin remodeling is context dependent rather than uniformly pro-longevity or pro-aging (Yu et al., 2025).

Beyond histone modifications, SCFAs also influence DNA methylation patterns and noncoding RNA networks. Butyrate-mediated HDAC inhibition enhances acetylation at promoter and enhancer regions of genes involved in immune tolerance and epithelial integrity, indirectly stabilizing DNA methylation landscapes that otherwise erode with age. Concurrently, SCFAs modulate the expression of microRNAs implicated in inflammatory signaling, mitochondrial biogenesis, and senescence-associated secretory phenotype (SASP), reinforcing their role as multifaceted regulators of epigenetic aging (Wang et al., 2022; Stein and Riber, 2023).

Tryptophan-derived microbial metabolites represent a second major class of epigenetically active compounds with relevance to aging. Indole derivatives generated by commensal bacteria activate the aryl hydrocarbon receptor (AhR), a ligand-dependent transcription factor that integrates microbial cues with host gene regulation. AhR signaling governs chromatin remodeling and transcriptional programs involved in immune cell polarization, barrier function, and inflammatory resolution. Dysregulation of this pathway during aging contributes to chronic low-grade inflammation and impaired immune surveillance, hallmarks of immunosenescence and inflammaging (Hezaveh et al., 2022; An et al., 2025). Importantly, sustained exposure to microbial indoles can induce durable transcriptional reprogramming in myeloid and epithelial cells, suggesting long-term epigenetic imprinting driven by microbial metabolism (Yang B. et al., 2025).

Emerging evidence also implicates microbiome-derived metabolites in age-associated clonal hematopoiesis and stem-cell dysfunction. Circulating bacterial intermediates that increase with age can activate intracellular pattern-recognition pathways, leading to transcriptional reprogramming and epigenetic alterations in hematopoietic stem and progenitor cells. These changes favor clonal expansion and skewed lineage commitment through NF-κB–dependent gene networks and altered chromatin states, linking microbial metabolic leakage to aging-related hematologic risk (Agarwal et al., 2025; Li X. et al., 2023).

Polyphenol-derived postbiotics, such as urolithins, further exemplify how microbial metabolism intersects with epigenetic control of aging. Generated through microbial biotransformation of dietary ellagitannins, urolithin A promotes mitophagy and mitochondrial gene expression via transcriptional activation of longevity-associated pathways. Such regulatory outcomes are accompanied by favorable reprogramming of inflammatory and metabolic gene networks, underscoring the ability of microbiome-derived metabolites to re-establish youthful transcriptional profiles within aging tissues (Yang Y. et al., 2025; Singh et al., 2022).

## Immune aging modulated by microbiome-gerogene signaling networks

Aging of the immune system is characterized by a progressive decline in immune competence (immunosenescence) accompanied by a persistent state of low-grade inflammation (inflammaging). Increasing evidence indicates that these processes are not solely driven by intrinsic immune cell exhaustion but are profoundly shaped by bidirectional signaling between the gut microbiome and host gerogenes, genes that regulate longevity, stress resistance, and cellular senescence. This microbiome-gerogene crosstalk emerges as a central regulatory network influencing immune aging trajectories and systemic healthspan (Caldarelli et al., 2024; Kawamoto and Hara, 2024; Khazi and Ahmad, 2025).

Age-associated remodeling of the gut microbiome leads to reduced microbial diversity, loss of beneficial taxa, and enrichment of proinflammatory or opportunistic species. These compositional shifts directly impact immune homeostasis by altering antigenic load, epithelial barrier integrity, and metabolite availability (Ramasinghe et al., 2025; Portincasa et al., 2024). Microbiota-driven increases in microbial translocation across a weakened intestinal barrier expose immune cells to chronic stimulation, promoting SASP and reinforcing inflammaging. In this context, gerogenic programs encompassing CDKN2A (p16^INK4a^), TP53, FOXO, and mTOR–AMPK regulatory axes integrate microbial and inflammatory cues, thereby modulating immune cell fate decisions toward exhaustion or dysfunctional activation (Bosco and Noti, 2021; Binyamin et al., 2025). Here, the term “gerogenes” is used to denote integrated signaling programs and transcriptional states rather than single gene effects, reflecting coordinated immune aging trajectories shaped by microbial and inflammatory cues.

A key mechanism linking microbiome alterations to immune aging involves B cell and T cell senescence. Chronic microbial stimulation in aged hosts has been shown to induce senescence in germinal center B cells, resulting in reduced immunoglobulin A (IgA) production and diversity. This impairs mucosal immune exclusion, further exacerbating dysbiosis and creating a self-reinforcing cycle between immune senescence and microbial imbalance (Yadav et al., 2025; Mizuno et al., 2024). At the transcriptional level, this process is accompanied by upregulation of cell-cycle arrest gerogenes and DNA damage response pathways, highlighting how microbial cues converge on conserved aging programs within adaptive immune cells (Bosco and Noti, 2021; Kawamoto and Hara, 2024). Recent evidence suggests that microbiome-driven immune remodeling is spatially organized and context dependent, with microbial signals differentially shaping immune surveillance, checkpoint activity, and myeloid polarization across distinct anatomical niches. This supports a model in which immune aging reflects localized failures in host-microbe regulation that propagate systemically, rather than a uniform decline in immune competence (Bautista et al., 2025d; Chao et al., 2025).

Microbial metabolites represent another critical axis of microbiome-gerogene-immune interaction. SCFAs, particularly butyrate and propionate, exert pleiotropic effects on immune aging by acting as epigenetic and metabolic regulators. SCFAs modulate histone acetylation and inhibit HDACs, influencing the expression of genes involved in T cell differentiation, effector function, and regulatory T cell (Treg) stability. In aged immune systems, reduced SCFA availability due to dysbiosis contributes to impaired Treg induction and heightened proinflammatory signaling, accelerating inflammaging (Best et al., 2025; Furusawa et al., 2013). Metabolite-driven regulatory signals converge with gerogene-controlled pathways that govern chromatin accessibility, mitochondrial dynamics, and cellular stress response programs (Tian et al., 2016).

Innate immunity is similarly shaped by microbiome-gerogene interactions during aging. Age-related dysbiosis promotes a myeloid-biased hematopoietic output, accompanied by epigenetic reprogramming of hematopoietic stem and progenitor cells. This skewing favors proinflammatory monocytes and dysfunctional macrophages with reduced phagocytic capacity but heightened cytokine secretion. Gerogenes regulating stem cell self-renewal, oxidative stress resistance, and metabolic flexibility act as molecular hubs integrating microbial-derived inflammatory signals into long-term innate immune remodeling (Caldarelli et al., 2024; Cisneros et al., 2022; Luu et al., 2018).

Systems-level analyses further reveal that aging is associated with a decline in beneficial host–microbiome metabolic interactions across multiple organs, including the gut, liver, and brain. Integrated metagenomic, transcriptomic, and metabolomic studies demonstrate that reduced microbial metabolic activity correlates with downregulation of host immune and barrier-related gene networks that are partly microbiome-dependent. Notably, immune-related host transcripts involved in antigen presentation, T cell proliferation, and inflammatory resolution show strong associations with microbial metabolic pathways, underscoring the role of microbiome-sensitive gerogenes in coordinating immune resilience across tissues (Binyamin et al., 2025; Heintz and Mair, 2014; Nguyen and Cho, 2025).

Importantly, immune aging is not uniformly detrimental and may include adaptive remodeling aimed at maintaining homeostasis under chronic stress. Centenarian-associated microbiome signatures and immune phenotypes suggest that specific microbial configurations can promote balanced inflammaging compatible with longevity. These beneficial states appear to rely on preserved microbial-gerogene signaling that restrains excessive inflammation while sustaining essential immune surveillance functions (Bosco and Noti, 2021; Dou et al., 2024).

## Gut barrier dysfunction and cellular senescence in systemic aging

The intestinal barrier represents a critical interface between the host and its external environment, integrating epithelial integrity, immune surveillance, and microbial homeostasis. Accumulating evidence indicates that age-associated disruption of this barrier is not merely a localized gastrointestinal phenomenon but a central driver of systemic aging and functional decline. Across species, intestinal barrier dysfunction emerges as an evolutionarily conserved hallmark of aging, tightly linked to microbial dysbiosis, chronic inflammation, cellular senescence, and increased mortality risk (Gieryńska et al., 2022; Salazar et al., 2018).

With advancing age, structural and functional alterations occur at multiple levels of the gut barrier. These include thinning of the mucus layer, reduced expression and mislocalization of tight junction proteins such as occludin and ZO-1, impaired antimicrobial peptide secretion, and dysregulation of intestinal stem cell (ISC) renewal. Taken together, such alterations heighten intestinal permeability, thereby enabling microbial translocation and systemic exposure to pathogen-associated molecular patterns (PAMPs), including lipopolysaccharide (LPS) (Salazar et al., 2023; Walrat et al., 2021; Zhang et al., 2025). Commonly described as increased intestinal permeability or “leaky gut,” this condition serves as a powerful initiator of persistent low-grade inflammation, a hallmark of the aging process.

Microbiome alterations play a central role in initiating and perpetuating barrier dysfunction. Age-associated shifts in microbial composition, characterized by reduced diversity and expansion of pathobionts, have been shown to directly compromise epithelial integrity and promote inflammatory signaling (Tseng and Wu, 2025; Thevaranjan et al., 2018) Experimental models demonstrate that germ-free or microbiota-depleted aged organisms are partially protected from systemic inflammation and immune dysfunction, underscoring the causal contribution of the microbiome to barrier breakdown and inflammaging (Jing et al., 2025). Conversely, fecal microbiota transplantation from young donors restores epithelial tight junction expression, reduces inflammatory cytokine production, and improves immune homeostasis in aged hosts (Jing et al., 2025; Abankwah et al., 2024).

Intestinal barrier failure is closely intertwined with cellular senescence at both local and systemic levels. Senescent epithelial and immune cells accumulate within the aging gut, exhibiting stable cell-cycle arrest and a senescence-associated secretory phenotype (SASP) enriched in pro-inflammatory cytokines, chemokines, and matrix-remodeling enzymes. This pro-inflammatory milieu exacerbates epithelial damage, impairs ISC regenerative capacity, and reinforces barrier dysfunction, establishing a self-sustaining vicious cycle. Notably, chronic intestinal inflammation, even when initiated earlier in life, accelerates senescent cell accumulation and promotes features of premature intestinal aging (Salazar et al., 2023).

At the molecular level, multiple stress-responsive pathways converge to link gut barrier dysfunction with senescence. Oxidative stress, mitochondrial dysfunction, and DNA damage responses are activated in aging intestinal epithelial cells, driving senescence through p53–p21 and p16^INK4a^ pathways (Ferreira-Gonzalez et al., 2025; Mansfield et al., 2024). Senescent cells further amplify inflammation through cGAS–STING activation in response to cytoplasmic DNA fragments, thereby extending local barrier defects into systemic inflammatory signaling. These processes align the aging gut with core hallmarks of aging, including altered intercellular communication and deregulated immune responses (Walrat et al., 2021; Guo et al., 2022).

Experimental evidence from invertebrate and mammalian models highlights the systemic consequences of intestinal barrier collapse. In *Drosophila*, loss of epithelial junction proteins leads to rapid microbial translocation, metabolic dysregulation, immune hyperactivation, and shortened lifespan, whereas restoration of junction integrity is sufficient to extend longevity. Similarly, aged mice exhibit increased intestinal permeability associated with elevated circulating TNF-α, IL-6, and IFN-γ, alongside impaired macrophage function and reduced pathogen clearance (Jing et al., 2025; Salazar et al., 2018; Salazar et al., 2023).

Importantly, recent studies suggest that gut barrier dysfunction is not an irreversible consequence of aging but a modifiable process. Interventions targeting microbial composition, epithelial junction stability, and inflammatory signaling have demonstrated promising geroprotective effects. Dietary strategies, time-restricted feeding, microbiota-targeted therapies, and senescence-modulating approaches can improve barrier integrity, reduce systemic inflammation, and partially restore immune competence in aged organisms (Tseng and Wu, 2025; Abankwah et al., 2024; Qin et al., 2025).

In the context of the microbiome–gerogene axis, gut barrier dysfunction serves as a mechanistic bridge linking microbial ecology to cellular senescence and systemic aging. Microbiota-driven alterations in epithelial signaling and immune activation modulate gerogene networks that govern stress resistance, inflammation, and tissue homeostasis. Persistent barrier failure therefore accelerates aging trajectories by amplifying senescence-associated inflammatory circuits across multiple organs (Thevaranjan et al., 2018; Guo et al., 2022). Intestinal barrier integrity thus emerges as a rate-limiting regulator of systemic aging rather than a secondary consequence, highlighting epithelial resilience as a strategic intervention point within the microbiome-gerogene axis.

## Microbiome regulation of mitochondrial function and metabolic gerogenes

Mitochondrial dysfunction is a central hallmark of aging and a key driver of metabolic gerogenes that regulate energy homeostasis, redox balance, and cellular resilience. Increasing evidence indicates that the gut microbiome acts as a critical upstream modulator of mitochondrial biology through metabolite-mediated signaling, immune-metabolic crosstalk, and host-microbe co-metabolism. This bidirectional interaction positions the microbiome as an essential regulator of mitochondrial function and, consequently, of aging trajectories governed by metabolic gerogenes (Cox et al., 2022; Ren et al., 2022).

One of the primary mechanisms by which the microbiome influences mitochondrial activity is through microbially derived metabolites that directly affect oxidative phosphorylation (OXPHOS), mitochondrial biogenesis, and mitophagy. SCFAs, particularly butyrate and propionate, serve not only as energy substrates but also as signaling molecules that enhance mitochondrial respiration efficiency and reduce electron leakage from the respiratory chain, thereby limiting excessive reactive oxygen species (ROS) generation (Li Y. et al., 2023; Zachos et al., 2024; Induri et al., 2022). Through activation of AMPK and PGC-1α pathways, SCFAs promote mitochondrial biogenesis and improve metabolic flexibility, processes that decline with age and are tightly linked to metabolic gerogene regulation (Ren et al., 2022; Andreux et al., 2019).

Beyond SCFAs, the microbiome regulates mitochondrial quality control through metabolites that modulate mitophagy, a geroprotective process essential for removing dysfunctional mitochondria. Urolithin A, a postbiotic produced by gut microbial metabolism of dietary ellagitannins, represents a paradigmatic example. Evidence from preclinical models and human studies indicates that urolithin A stimulates mitophagic pathways, promotes mitochondrial renewal, and enhances muscular performance and metabolic efficiency in middle-aged and older individuals, supporting a direct and translationally relevant microbiome-mitochondria-gerogene axis (Singh et al., 2022; Xu X. et al., 2025).

Redox homeostasis represents another critical node connecting the microbiome to mitochondrial gerogenes. Age-related dysbiosis is associated with increased intestinal permeability and systemic exposure to microbial products such as LPS, which induce mitochondrial ROS production and oxidative stress in peripheral tissues (Thevaranjan et al., 2017; Abahussin et al., 2025). Excessive ROS not only damages mitochondrial DNA (mtDNA) but also activates inflammatory signaling pathways that reinforce metabolic dysfunction and cellular senescence. Conversely, a balanced microbiome supports antioxidant defenses by regulating NAD^+^/NADH and NADP^+^/NADPH pools, thereby preserving mitochondrial redox capacity and genomic stability (Yusri et al., 2025).

NAD^+^ metabolism is particularly relevant in this context, as NAD^+^ is a central cofactor for mitochondrial respiration, sirtuin activity, and DNA repair, core processes governed by metabolic gerogenes. Gut microbes influence host NAD^+^ availability primarily through modulation of NAD^+^ precursor pools, inflammatory tone, and host salvage pathways, thereby indirectly shaping mitochondrial energy production and cellular stress resistance (Qiao et al., 2024). Age-associated declines in NAD^+^ levels have been linked to impaired mitophagy, reduced ATP generation, and increased susceptibility to metabolic and neurodegenerative disorders, positioning the microbiome as a modifiable upstream determinant of this gerogenic pathway (Andreux et al., 2019; Prolla and Denu, 2014). Microbial regulation of NAD^+^ homeostasis further operates via inflammation-driven NAD^+^ consumption and altered redox balance, collectively shaping mitochondrial resilience during aging.

Host mitochondrial genotype and functional status also exert reciprocal control over gut microbial ecology, establishing a bidirectional regulatory circuit. Alterations in mitochondrial reactive oxygen species production modulate microbial diversity and community structure, indicating that mitochondrial genetics and age-associated mitochondrial deterioration can actively promote dysbiosis (Yardeni et al., 2019). Such reciprocal reinforcement between mitochondrial dysfunction and microbiome imbalance likely intensifies metabolic gerogene dysregulation and accelerates systemic aging trajectories (Bubb et al., 2025).

The microbiome also interfaces with pharmacological modulators of mitochondrial gerogenes. Metformin, a widely used geroprotective drug, exerts many of its metabolic and anti-aging effects through the gut microbiome, where it alters microbial composition and metabolite production (Wang et al., 2024a). Microbiome-dependent regulatory influences promote enhanced mitochondrial efficiency, attenuated inflammatory signaling, and greater metabolic resilience, reinforcing the concept that mitochondrial gerogenes operate within an interconnected, microbiome-shaped metabolic network (Ramasinghe et al., 2025; Zachos et al., 2024).

Current evidence supports a framework in which the gut microbiome acts as an important upstream modulator of mitochondrial integrity by shaping cellular energy metabolism, redox homeostasis, and mitochondrial quality control. Microbiome-derived metabolites, including short-chain fatty acids, urolithin A, and NAD^+^-associated intermediates, influence metabolic gerogenes that govern cellular longevity and systemic aging trajectories. Perturbation of this regulatory axis through dysbiosis favors mitochondrial dysfunction, inflammaging, and metabolic deterioration, whereas targeted microbiome-based strategies provide a rational avenue to restore mitochondrial fitness and support healthy aging (McBeth et al., 2025; Fujisaka et al., 2023).

## Dietary, hormonal, and lifestyle determinants of microbiome-gerogene interactions

Dietary patterns, hormonal status, and lifestyle behaviors represent highly plastic determinants shaping the microbiome-gerogene axis across the lifespan. Interacting environmental and host-related determinants shape microbial community structure, metabolite output, endocrine crosstalk, and epigenetic regulation, thereby modulating central gerogenes involved in inflammaging, metabolic deterioration, and progressive loss of physiological resilience.

### Dietary modulation of microbial-gerogene signaling

Diet is among the most potent regulators of gut microbial composition and function. Diets enriched in dietary fiber, whole grains, fruits, vegetables, and phytochemicals consistently promote microbial diversity and favor taxa associated with SCFA production, including butyrate and propionate producers (Fujisaka et al., 2023; Berding et al., 2021). These SCFAs function as bioenergetic substrates and as signaling molecules capable of directly modifying chromatin through histone acylation, thereby influencing transcriptional programs governing mitochondrial function, stress resistance, and cellular differentiation. Through such mechanistic pathways, diet-derived microbial metabolites impose regulatory control over gerogenes governing nutrient-sensing circuits (AMPK-mTOR), redox homeostasis, and epigenetic stability (Nshanian et al., 2025; Xu Z. et al., 2025).

In contrast, Westernized dietary patterns characterized by excess refined carbohydrates, saturated fats, alcohol, and salt intake induce dysbiosis marked by reduced SCFA production and increased endotoxin-producing taxa (Kang et al., 2023; Malesza et al., 2021). This shift promotes low-grade systemic inflammation and metabolic endotoxemia, both of which are known activators of pro-aging gene networks linked to insulin resistance, immune dysfunction, and vascular aging. Importantly, interindividual variability in the capacity to convert dietary phytochemicals into bioactive metabolites, such as urolithins, equol, or sulforaphane derivatives, further explains heterogeneity in gerogene responsiveness to identical dietary exposures (Mohammad and Thiemermann, 2020; Beaver et al., 2025).

### Hormonal regulation and sexual dimorphism in microbiome-gerogene interactions

Sex hormones represent a critical endogenous layer shaping microbiome composition and function. Estrogens, androgens, and progesterone influence gut barrier integrity, bile acid metabolism, immune tone, and microbial enzymatic activity, collectively contributing to sex-specific microbial signatures. The gut microbiota reciprocally modulates systemic hormone levels through deconjugation and enterohepatic recirculation, particularly via the estrobolome, thereby creating a bidirectional regulatory loop with direct relevance for aging trajectories (Zommiti and Feuilloley, 2025; Santos-Marcos et al., 2023).

Hormonal transitions such as menopause and age-related reproductive decline are accompanied by shifts in microbiome–host endocrine interactions that extend to organ-level regulation. Experimental evidence shows that gut microbiota modulate ovarian function during aging. Transplantation of microbiota from reproductively aged donors into young recipients induces impaired ovarian function, whereas transfer from hormonally preserved donors restores follicular development, endocrine profiles, and oocyte quality (Kim et al., 2026).

Microbial communities regulate estrogen availability through deconjugation and enterohepatic recirculation and alter circulating metabolites linked to mitochondrial activity, oxidative balance, and steroidogenic capacity in ovarian tissue. Microbiome-associated inflammatory signaling disrupts ovarian niche homeostasis, supporting a link between dysbiosis and accelerated reproductive aging. The gut microbiome operates as a regulator of the hypothalamic–pituitary–gonadal axis, with effects extending from hormone metabolism to tissue-level control of endocrine aging (Kim et al., 2026; Wang H. et al., 2025).

### Lifestyle factors as amplifiers or suppressors of gerogenic signaling

Beyond diet and hormones, lifestyle behaviors such as physical activity, sleep regularity, stress exposure, medication use, and environmental context exert profound effects on microbiome–gerogene dynamics. Regular physical activity is associated with increased microbial diversity and enrichment of SCFA-producing taxa, contributing to improved mitochondrial efficiency, anti-inflammatory signaling, and epigenetic stability (Fujisaka et al., 2023). Conversely, sedentary behavior, chronic psychosocial stress, and circadian disruption promote dysbiosis and neuroendocrine imbalance, reinforcing inflammaging circuits (You et al., 2022). Circadian misalignment induced by shift work, irregular sleep, or mistimed feeding can simultaneously desynchronize peripheral metabolic programs and disrupt microbial oscillations, amplifying low-grade inflammation and metabolic stress. Conversely, chrononutrition approaches such as time-restricted eating/feeding can restore microbiota rhythmicity and strengthen oscillations in SCFA and bile-acid pathways, thereby improving metabolic readouts in a clock-aligned manner (Bautista et al., 2025f; Bautista and López-Cortés, 2025a; Alum, 2025).

Pharmacological exposures frequently associated with modern lifestyles further modulate this axis. Metformin, for instance, reshapes microbial composition toward taxa linked to improved metabolic health and longevity, suggesting that part of its geroprotective effects may be microbiome-mediated rather than solely host-centric. Similarly, probiotics and fermented foods have demonstrated the capacity to modulate conserved longevity pathways in experimental systems, including insulin/IGF-1 and FOXO-related signaling cascades, supporting the concept that targeted microbial interventions can influence gerogene activity (Induri et al., 2022; Ding et al., 2025; Kumaree et al., 2023).

Dietary quality, hormonal context, and lifestyle behaviors function as tightly linked regulators of microbial ecology and its downstream signaling capacity. Microbiome-derived metabolites, endocrine feedback loops, and epigenetic mechanisms transmit these environmental inputs to gerogenes that shape the tempo of biological aging and the maintenance of healthspan. Recognition of this layered regulatory architecture provides a foundation for precision geromedicine approaches that integrate personalized nutrition, hormonal assessment, and lifestyle modulation to reprogram the microbiome-gerogene axis in favor of physiological resilience and healthy aging (García Cobarro et al., 2025; Rubas et al., 2025; Kadyan et al., 2025).

## Gut microbiome contributions to the hallmarks of aging and age-related diseases

**FIGURE 1:**
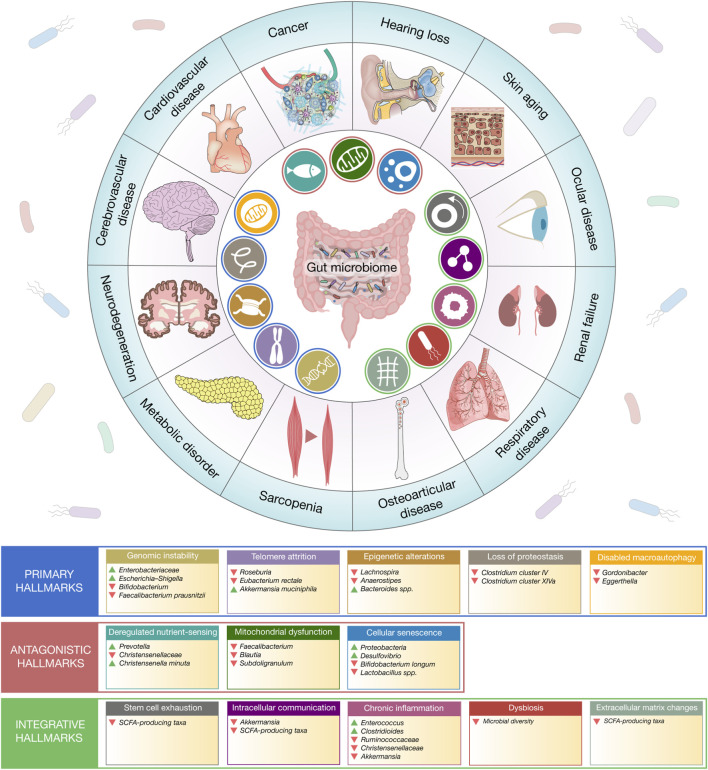
The gut microbiome–gerogene axis as a central regulator of ageing-associated diseases. Schematic overview illustrating how the gut microbiome functions as a systemic modulator of the molecular hallmarks of ageing (“gerogenes”) and their downstream contribution to multisystem disease. The central panel depicts the gut microbiome as a hub integrating microbial communities, metabolites, and host–microbe signalling pathways. Surrounding icons represent core ageing-associated molecular processes, including chronic inflammation, dysbiosis, stem cell exhaustion, genomic instability, telomere attrition, epigenetic alterations, loss of proteostasis, disabled macroautophagy, mitochondrial dysfunction, deregulated nutrient sensing, cellular senescence, and altered intracellular communication. For each hallmark, representative microbial taxa with reported mechanistic or associative links are highlighted. The outer ring illustrates major organ-specific and systemic diseases influenced by microbiome-driven ageing pathways, including cancer, neurodegeneration, cerebrovascular and cardiovascular diseases, metabolic disorders, sarcopenia, osteoarticular disease, respiratory disease, renal failure, ocular disease, skin ageing, and hearing loss. Collectively, the figure emphasizes that microbiome-derived signals act upstream of multiple gerogenic processes, coordinating molecular ageing trajectories across tissues and contributing to age-related functional decline and disease susceptibility.

Aging arises from the progressive accumulation of molecular damage, impaired stress responses, and systemic dysregulation, conceptualized through twelve interconnected hallmarks. Evidence supports a role for the gut microbiome as an upstream regulator of these processes, influencing the trajectory of age-related decline and disease susceptibility. Microbial communities modulate genomic stability, metabolic homeostasis, immune function, and tissue integrity, linking intestinal ecology to organismal aging (López-Otín et al., 2023a; Kanimozhi and Sukumar, 2025; Simbirtseva and O’Toole, 2025). To improve interpretability, hallmarks are organized into primary, antagonistic, and integrative categories, reflecting early drivers of damage, compensatory responses, and system-level outcomes (Figure 1) (López-Otín et al., 2023b).

### Primary hallmarks

Primary hallmarks include molecular alterations that initiate aging processes. Genomic instability is influenced by microbiome-associated inflammatory and metabolic stressors. Age-related dysbiosis, characterized by enrichment of *Enterobacteriaceae* and *Escherichia-Shigella*, increases oxidative stress and DNA damage signaling, contributing to mutation accumulation in proliferative tissues. Commensal taxa such as *Bifidobacterium* and *Faecalibacterium prausnitzii* support redox balance through SCFA production and contribute to DNA repair pathways (López-Otín et al., 2023a; Aldriwesh et al., 2026; Simbirtseva and O’Toole, 2025).

Telomere attrition is associated with microbial regulation of inflammation and oxidative stress. Reduced abundance of butyrate-producing taxa, including *Roseburia* and *Eubacterium rectale*, correlates with shortened leukocyte telomere length in metabolic and cardiovascular disease contexts. *Akkermansia muciniphila* has been linked to preserved telomere dynamics through improved metabolic control and reduced endotoxemia (Guo et al., 2022; Kanimozhi and Sukumar, 2025; Bradley and Haran, 2024).

Epigenetic alterations represent a major interface between microbial metabolism and host gene regulation. Microbial metabolites such as butyrate, propionate, folate, and secondary bile acids influence histone acetylation and DNA methylation. Reduced abundance of SCFA-producing genera, including *Lachnospira* and *Anaerostipes*, is associated with epigenetic dysregulation in neurodegenerative diseases, whereas enrichment of *Bacteroides* species is linked to inflammatory epigenetic remodeling in metabolic disorders (Guo et al., 2022; Bradley and Haran, 2024; Borrego-Ruiz and Borrego, 2024).

Loss of proteostasis is influenced by microbial metabolic outputs that regulate protein folding, degradation, and quality control systems. Depletion of SCFA-producing taxa, particularly *Clostridium* clusters IV and XIVa, reduces SCFA-mediated proteasomal activity and impairs protein turnover, facilitating accumulation of misfolded proteins in neurodegenerative and aging-associated conditions. Microbial-derived metabolites also modulate chaperone expression and ubiquitin–proteasome pathways, linking intestinal ecology to systemic proteostasis maintenance (Borrego-Ruiz and Borrego, 2024; DeJong et al., 2020).

Disabled macroautophagy reflects impaired autophagic and mitophagic processes associated with altered microbial metabolism. Reduced availability of microbial metabolites, including polyamines and urolithin A, compromises autophagic flux and mitochondrial quality control. Taxa such as *Gordonibacter* and *Eggerthella*, involved in urolithin production, are associated with enhanced mitophagy and preservation of cellular homeostasis. Disruption of these microbial pathways contributes to defective organelle turnover and accumulation of cellular damage during aging (Borsky et al., 2025; Pradeepkiran et al., 2022).

### Antagonistic hallmarks

Antagonistic hallmarks arise as adaptive responses to primary damage but become detrimental under persistent activation. Deregulated nutrient sensing reflects interactions between microbial composition and host metabolic signaling. Microbiome-associated shifts influence insulin–IGF-1 and mTOR pathways, contributing to metabolic dysfunction. Expansion of *Prevotella* and reduction of *Christensenellaceae* are associated with obesity and insulin resistance, whereas enrichment of *Christensenella minuta* is observed in metabolically stable individuals and long-lived populations (Bradley and Haran, 2024; Kong et al., 2016).

Mitochondrial dysfunction is shaped by microbial regulation of redox balance and energy metabolism. Reduced abundance of SCFA-producing taxa is associated with decreased NAD^+^ availability and impaired mitochondrial biogenesis. In contrast, enrichment of *Faecalibacterium*, *Blautia*, and *Subdoligranulum* supports mitochondrial function through metabolite-mediated signaling (Simbirtseva and O’Toole, 2025; Borrego-Ruiz and Borrego, 2024).

Cellular senescence is reinforced by microbiome-driven inflammatory signaling and metabolite imbalance. Expansion of proinflammatory taxa such as *Proteobacteria* and *Desulfovibrio* promotes senescence-associated secretory phenotype, sustaining cytokine production and tissue dysfunction. Microbial configurations enriched in *Bifidobacterium longum* and *Lactobacillus* species are associated with reduced inflammatory tone and modulation of senescence-associated pathways (Kawamoto and Hara, 2024; Jang et al., 2024).

### Integrative hallmarks

Integrative hallmarks reflect cumulative system-level effects of aging. Stem cell exhaustion is linked to chronic microbiome-mediated inflammation and impaired niche signaling. Persistent dysbiosis alters regenerative capacity through cytokine-driven disruption of stem cell maintenance, whereas microbial communities enriched in SCFA-producing taxa support epithelial renewal and hematopoietic function (Simbirtseva and O’Toole, 2025; Tenchov et al., 2024).

Altered intercellular communication arises from microbiome-dependent regulation of immune, endocrine, and metabolic signaling. Loss of microbial diversity is associated with increased systemic inflammation through LPS-mediated Toll-like receptor activation, contributing to vascular, renal, and neurodegenerative disease processes. Microbial communities enriched in *Akkermansia* and SCFA-producing taxa are associated with improved immune coordination and signaling homeostasis (Aldriwesh et al., 2026; Kanimozhi and Sukumar, 2025; Tenchov et al., 2024).

Chronic inflammation represents a central integrative hallmark linking microbial dysbiosis to systemic aging. Persistent enrichment of pathobionts such as *Enterococcus* and *Clostridioides* is associated with sustained low-grade inflammation and progression of age-related diseases. Microbiomes observed in long-lived populations, enriched in *Ruminococcaceae*, *Christensenellaceae*, and *Akkermansia*, are associated with anti-inflammatory functional profiles and preserved healthspan (Guo et al., 2022; Bradley and Haran, 2024; Kong et al., 2016).

Dysbiosis constitutes an integrative hallmark that both drives and reflects age-associated physiological decline. Age-related restructuring of microbial communities reduces functional diversity, alters metabolite production, and disrupts host–microbe homeostasis, amplifying inflammatory, metabolic, and immune dysregulation across tissues (Zermeño-Ruiz et al., 2026; Sajid et al., 2026).

Extracellular matrix changes are influenced by microbiome-dependent regulation of inflammation and tissue remodeling pathways. Microbial metabolites and inflammatory mediators modulate fibroblast activity, collagen deposition, and matrix degradation, contributing to fibrosis and loss of tissue elasticity in aging organs (Selman and Pardo, 2021; Mayorca-Guiliani et al., 2025).

Available evidence supports a framework in which the gut microbiome operates as a regulator across multiple aging hallmarks, integrating environmental and host-derived signals into molecular pathways that shape aging trajectories. Associations between microbial composition and aging phenotypes are influenced by diet, medication, geography, and methodological variability, highlighting the importance of functional and metabolomic profiling. Longitudinal and interventional studies are required to establish causal relationships in human populations.

## Microbiome-based precision interventions for gerogene regulation

The conceptual transition from descriptive geroscience to actionable precision geromedicine has positioned the gut microbiome as a modifiable upstream regulator of gerogenes, genes and pathways that accelerate biological aging. Accumulating evidence indicates that targeted manipulation of microbial composition and function can recalibrate gerogenic signaling networks, thereby attenuating inflammaging, preserving barrier integrity, and restoring systemic homeostasis. Microbiome-based precision interventions therefore represent a mechanistically grounded strategy to modulate aging trajectories in a personalized manner (Parker et al., 2022; Kroemer et al., 2025b; Bautista et al., 2026a).

Among currently explored interventions, fecal microbiota transplantation (FMT) is the strongest experimental evidence between microbial ecology and gerogene regulation. Preclinical studies consistently show that transfer of microbiota from young donors to aged recipients reverses hallmarks of aging across multiple organs, including restoration of intestinal barrier function, reduction of systemic pro-inflammatory cytokines, and normalization of age-associated transcriptional programs in immune and neural tissues. These effects are tightly linked to downregulation of gerogenic pathways such as NF-κB, type I interferon signaling, and stress-responsive transcriptional networks, highlighting the microbiome as an upstream controller of inflammatory gerogenes rather than a passive correlate of aging (Jing et al., 2025; Novelle et al., 2025). While these findings provide strong proof-of-concept in preclinical systems, controlled human studies with standardized donor screening, strain-level tracking, and long-term follow-up remain limited. Establishing durability, safety, and reproducible gerogene-linked endpoints in older and frail populations will be essential before FMT can be positioned as a scalable gerotherapeutic strategy.

Beyond whole-community transfer, precision probiotic and next-generation live biotherapeutic products (LBPs) enable strain-level modulation of gerogene-associated pathways. Specific taxa, including *A. muciniphila*, *Bifidobacterium* spp., and members of *Christensenellaceae*, have been shown to reinforce tight-junction integrity, reduce endotoxemia, and suppress pro-inflammatory gerogenic cytokines such as IL-6, IL-17, and IFN-γ (Jing et al., 2025). Convergent regulatory outcomes engage conserved aging pathways, including insulin/IGF-1 signaling, AMPK activation, and mitochondrial stress response programs, positioning defined microbial consortia as tunable modulators of metabolic and immune gerogenes (Kroemer et al., 2025a; Shi et al., 2024).

Diet-driven microbiome modulation represents a complementary and scalable precision intervention. Nutritional patterns rich in fermentable fibers, polyphenols, and diverse plant-derived bioactives selectively enrich SCFA producing microbes, leading to increased butyrate, propionate, and acetate availability. These microbial metabolites act as epigenetic and transcriptional modulators, inhibiting HDACs, dampening chronic inflammatory signaling, and promoting gerosuppressive programs linked to mitochondrial efficiency and stress resilience. Importantly, emerging frameworks recognize “nutrition dark matter”, previously uncharacterized food-derived small molecules, as substrates for microbial biotransformation into bioactive compounds capable of influencing gerogene expression in a context-dependent manner (Shi et al., 2024; Montserrat-de la Paz, 2021; Carlberg et al., 2025).

Advanced microbiome-based therapeutics extend beyond diet and probiotics to include engineered microbes, phage-based strategies, and targeted ecosystem editing. Metagenomic engineering approaches allow the rational design of microbial strains capable of producing specific metabolites or degrading pro-gerogenic compounds, thereby exerting predictable effects on host aging pathways (Montserrat-de la Paz, 2021; Nazir et al., 2024; Bautista and López-Cortés, 2026). In parallel, bacteriophage-mediated modulation of pathobionts offers a precision tool to suppress taxa that disproportionately contribute to inflammaging without disrupting overall community stability (Gulliver et al., 2024; Hitch et al., 2022). Differences in baseline ecology can shift colonization success, metabolite production, and immune modulation, helping explain why the same probiotic or diet-based strategy may yield divergent outcomes across cohorts. In parallel, technical heterogeneity across sampling, sequencing platforms, and bioinformatic pipelines can generate inconsistent taxonomic and functional profiles, reinforcing the need for predictive host-microbial biomarkers and rigorous standardization for reproducibility (Van Hul and Cani, 2026; Wei et al., 2025).

An essential attribute of microbiome-based precision interventions lies in their alignment with multi-omics biomarkers of aging. Integrated transcriptomic, metabolomic, and immunological analyses demonstrate that targeted microbiota remodeling is tightly linked to coordinated shifts in gerogene expression across the intestinal epithelium, lymphoid compartments, and distal organs, including the brain. Restoration of microbial diversity and functional capacity is associated with normalization of cellular stress-response programs, enhanced epithelial repair, and attenuation of SASP (Jing et al., 2025; Loh et al., 2024). Such evidence supports the application of microbiome-derived signatures both as actionable therapeutic targets and as dynamic indicators of intervention efficacy (Auclair-Ouellet et al., 2025).

Despite their promise, microbiome-based gerotherapeutics raise critical safety and regulatory considerations. Risks include horizontal transfer of antimicrobial resistance genes, unintended pathobiont expansion, and long-term ecological instability, particularly in frail or immunocompromised populations. Rigorous donor screening, strain-level characterization, and extended clinical follow-up will be essential to ensure safety and durability of microbiome-targeted interventions (Coyte et al., 2022; Chatzigiannidou et al., 2025).

Translating microbiome-based strategies into clinical geromedicine requires frameworks that explicitly address host heterogeneity, microbial ecosystem dynamics, and long-term safety. Individual factors such as age, sex, diet, prior antibiotic exposure, and baseline microbiome composition critically determine therapeutic responsiveness, supporting the need for stratified and adaptive intervention designs rather than uniform approaches (Kroemer et al., 2025a; Hitch et al., 2022). Sustainable benefits further depend on ecological resilience and functional stability of microbial communities, as transient or poorly integrated perturbations may limit efficacy or induce adverse effects. These complexities also challenge current regulatory models, which remain largely optimized for conventional pharmacological agents, highlighting the necessity for updated regulatory paradigms capable of evaluating live biotherapeutics and ecosystem-level interventions within precision aging and geromedicine frameworks (Kadyan et al., 2025; Gulliver et al., 2024).

## Limitations and sources of heterogeneity

Interpretation of the microbiome–gerogene axis is constrained by interindividual variability driven by environmental and host-related factors. Microbiome composition is shaped by diet, lifestyle, medication use, and socio-cultural context, resulting in heterogeneous baseline configurations across populations. Geographic and dietary patterns further contribute to variability, with distinct microbial structures observed across regions, limiting cross-cohort comparability and reducing reproducibility of aging-associated microbial signatures (Kanimozhi and Sukumar, 2025; Borrego-Ruiz and Borrego, 2024).

Aging-related microbiome changes do not follow a uniform trajectory. Differences between healthy aging, frailty, and extreme longevity indicate that microbial configurations vary according to host condition rather than chronological age alone. Some cohorts exhibit reduced diversity and enrichment of pathobionts, whereas long-lived individuals display distinct microbial profiles with unique functional characteristics, indicating that microbiome remodeling may reflect context-dependent or adaptive processes rather than a linear deterioration model (Simbirtseva and O’Toole, 2025).

A major limitation is the difficulty in establishing causality. Most available evidence derives from cross-sectional human studies, where microbial alterations are associated with aging phenotypes but lack temporal resolution to determine directionality (Bradley and Haran, 2024; Bautista et al., 2026c). Age-related changes in host physiology, including immunosenescence, intestinal barrier dysfunction, and chronic inflammation, can independently drive microbial shifts, complicating the distinction between cause and consequence. Experimental studies in animal models demonstrate that microbiome manipulation can influence aging-related outcomes, but translation to human systems remains limited (DeJong et al., 2020; O’Toole, 2024).

Confounding factors represent an additional source of heterogeneity. Microbiome composition in older individuals is strongly influenced by non-aging variables such as antibiotic exposure, polypharmacy, comorbidities, and dietary patterns, all of which independently alter microbial ecosystems (Kanimozhi and Sukumar, 2025; O’Toole, 2024). These factors frequently co-occur in aging populations, complicating attribution of observed microbial changes specifically to aging processes. Early-life exposures and long-term host–microbe interactions also contribute to persistent microbiome configurations that are rarely controlled for in cohort studies (Simbirtseva and O’Toole, 2025).

Methodological variability further limits reproducibility. Different sequencing approaches generate distinct representations of microbial communities, with 16S rRNA profiling providing limited taxonomic resolution and shotgun metagenomics offering broader functional insight but introducing additional analytical complexity. Variability in sample collection, processing, and bioinformatic pipelines introduces systematic bias, hindering cross-study integration and meta-analysis (Bradley and Haran, 2024; Wang et al., 2024b).

Functional interpretation remains incomplete. Taxonomic composition does not directly reflect microbial activity, and similar microbial profiles can produce divergent metabolomic outputs depending on environmental context and host physiology. Microbial metabolites, including short-chain fatty acids and bile acids, regulate host signaling pathways involved in aging, yet direct mechanistic links between microbial taxa, metabolite production, and downstream molecular pathways remain insufficiently defined (Borrego-Ruiz and Borrego, 2024; Xiao et al., 2025; Bautista et al., 2026b).

Longitudinal evidence is limited. Most studies rely on cross-sectional designs that capture interindividual variability but fail to resolve intraindividual temporal dynamics during aging. Lack of longitudinal sampling restricts identification of predictive microbial trajectories and limits causal inference (Wang L. et al., 2025).

The concept of a “healthy” microbiome in aging remains undefined. Microbial configurations associated with longevity differ from those observed in younger individuals, indicating that successful aging may involve distinct ecological states rather than preservation of a youthful microbiome (Wilmanski et al., 2022).

## Conclusions and future perspectives

Aging cannot be explained solely by intrinsic genetic programs but arises from bidirectional interactions between host gerogenic networks and the gut microbiome, constituting an integrated regulatory axis with systemic impact. Experimental and multi-omics evidence indicates that the host actively structures microbial ecology through intestine-specific genetic, immunological, and metabolic pathways, rather than acting as a passive habitat. Epithelial barrier integrity, antimicrobial defense systems, and nutrient-sensing circuits impose selective constraints on microbial communities, limiting dysbiosis and modulating downstream inflammatory and metabolic processes associated with aging (Dey, 2025). This host-centric perspective reframes the microbiome as a responsive biological system embedded within gerogenic regulation.

Across the lifespan, age-associated remodeling of the gut microbiome contributes to hallmark processes of aging, including inflammaging, immunosenescence, mitochondrial dysfunction, epigenetic drift, and stem cell exhaustion. Experimental and population-based studies consistently demonstrate that microbial dysbiosis increases intestinal permeability, promotes chronic low-grade inflammation, and impairs innate and adaptive immune functions, collectively accelerating biological aging and age-related morbidity (Bosco and Noti, 2021; Thevaranjan et al., 2017). Conversely, preserved microbial diversity and compositional adaptability in older individuals are associated with improved metabolic resilience, reduced frailty, and enhanced survival, supporting a contributory role of the microbiome in healthy aging, while acknowledging that causality remains context-dependent (Yardeni et al., 2019).

Mechanistically, the microbiome-gerogene axis operates through multiple converging layers. Microbial metabolites, including short-chain fatty acids, bile acid derivatives, and amino acid metabolites, interface with host epigenetic regulators, mitochondrial signaling pathways, and immune checkpoints to modulate gene expression programs linked to longevity and tissue repair. Epigenetic alterations, now recognized as partially reversible drivers of aging, are particularly sensitive to microbial inputs, positioning the microbiome as an upstream modulator of epigenetic clocks and transcriptional aging signatures (An et al., 2025). In parallel, mitochondrial function emerges as a critical node of host-microbe crosstalk: host mitochondrial genotype and redox status influence microbial community structure, while microbial metabolites reciprocally regulate mitochondrial bioenergetics, oxidative stress, and mitophagy, creating feedback loops that shape systemic aging phenotypes (Kawamoto and Hara, 2024; Wilman et al., 2021). Multilayered interactions parallel conserved principles of tissue conditioning and niche permissiveness, indicating that aging trajectories are shaped by spatially resolved host–microbiome dynamics rather than uniform chronological decline (Bautista and López-Cortés, 2025b).

A recurrent mechanism involves self-reinforcing interactions between gut dysbiosis and cellular senescence. Age-associated barrier dysfunction permits microbial translocation, promoting senescence-associated secretory phenotype (SASP) in epithelial and immune compartments. Resulting inflammatory signaling disrupts mucosal immunity and impairs IgA-mediated microbial containment, amplifying dysbiosis and accelerating both intestinal and systemic aging (Bosco and Noti, 2021; Thevaranjan et al., 2017). Interruption of this feedback loop remains a major challenge and a critical target for gerotherapeutic intervention.

From a translational perspective, the evidence supports a shift toward microbiome-informed precision geromedicine. Interventions targeting the microbiome–gerogene axis, including personalized nutrition, prebiotics, probiotics, postbiotics, fecal microbiota transplantation, senolytics, and mitochondrial-targeted therapies, offer potential to recalibrate aging trajectories. Embedding these approaches within circadian precision frameworks (chronotherapy/chrononutrition) may be particularly impactful, given that feeding time can realign microbial oscillations and metabolite rhythmicity even under clock disruption, supporting time-based personalization as part of geromedicine pipelines (Bautista and López-Cortés, 2025a; Franzago et al., 2023). Importantly, emerging evidence suggests that the efficacy of such interventions is strongly modulated by host genetic background, baseline microbiome configuration, immune status, and lifestyle factors, emphasizing the need for stratified and adaptive treatment frameworks (Dato et al., 2017; Yardeni et al., 2019). Integration of multi-omics profiling, including metagenomics, epigenomics, metabolomics, and mitochondrial genomics, will be essential to identify responsive subpopulations and define robust biomarkers of intervention success.

Future research should prioritize several directions. First, longitudinal and interventional human studies are needed to establish causality and temporal dynamics within the microbiome-gerogene axis, moving beyond associative findings. Second, tissue-specific and cell-type resolved models, including conditional genetic systems and single-cell multi-omics, will be critical to dissect localized host-microbe interactions that drive systemic aging (Dey, 2025; Xu X. et al., 2025). Third, regulatory and ethical frameworks must evolve to accommodate live biotherapeutics and ecosystem level interventions, ensuring long-term safety, reproducibility, and equitable access (Abankwah et al., 2024).

In conclusion, the microbiome-gerogene axis provides a unifying framework linking molecular hallmarks of aging with environmental and lifestyle exposures. By positioning the gut microbiome as an active regulator of gerogenic pathways, this paradigm opens new avenues for predictive, preventive, and personalized strategies aimed at extending healthspan rather than merely treating age-related diseases. Harnessing this axis through integrative, systems-level approaches represents a promising frontier for the next-generation of precision geromedicine.
